# Dual native G‐quadruplex folding is associated with chromatin looping at the *MYC* locus

**DOI:** 10.1002/2211-5463.70316

**Published:** 2026-07-23

**Authors:** Dieila Giomo de Lima, Gustavo Narvaes Guimarães, Emilyane de Oliveira Santana Amaral, Bianca Caroline Figueiredo Bianco, Manuel Jara‐Espejo, Sérgio Roberto Peres Line, Ana Paula de Souza, Aline Cristiane Planello

**Affiliations:** ^1^ Department of Biosciences Faculdade de Odontologia de Piracicaba/Universidade de Campinas (UNICAMP) Brazil; ^2^ Life Sciences Core Facility (LaCTAD) Universidade de Campinas (UNICAMP) Brazil; ^3^ Vall d'Hebron Institute of Oncology (VHIO) Barcelona Spain; ^4^ Department of Morphology and Pathology Faculdade de Medicina de Jundiaí (FMJ) Brazil

**Keywords:** chromatin looping, enhancer, epigenetics, G‐quadruplex, *MYC*, *PVT1*

## Abstract

DNA can adopt noncanonical four‐stranded structures known as G‐quadruplexes (G4s), which are enriched at regulatory regions and implicated in transcriptional control. However, the process by which native G4 folding contributes to enhancer–promoter communication and three‐dimensional genome organization remains poorly understood. Here, we investigated the relationship between G4 folding, chromatin state and long‐range regulatory interactions by integrating chromatin state maps, DNA methylation profiles, and BG4 ChIP‐seq data across two closely related human keratinocyte models. To assess long‐range regulatory contacts directly, we applied locus‐specific chromosome conformation capture (3C‐qPCR) at endogenous regulatory loci. Genome‐wide analyses revealed that both folded and unfolded G4 loci, operationally defined by BG4 detection, are enriched at transcription start sites and active enhancer regions. However, unfolded G4s preferentially associate with weak enhancers, consistent with a poised regulatory state. Focusing on the endogenous *MYC*–*PVT1* locus, enhancer–promoter contacts were detectable only in the cellular context where folded G4s were present at both the promoter and distal enhancers, independently of active histone marks alone. Together, these findings position this dual G4 folding as a genomic feature associated with enhancer–promoter communication and three‐dimensional regulatory architecture.

## Graphical abstract

BG4‐detectable G‐quadruplex (G4) in HaCaT and NHEK keratinocytes identified folded and unfolded G4s enriched at promoters/TSSs and active enhancers, whereas unfolded G4s also overlapped weak/poised enhancers. At *MYC*–*PVT1*, 3C‐qPCR detected enhancer–promoter looping only when G4s were simultaneously folded at both regulatory elements under native chromatin conditions. Created in biorender. De Lima, D. (2026) https://BioRender.com/2e0n5cm.

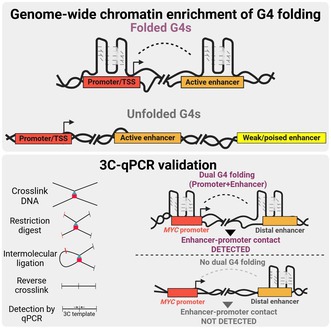

**Abbreviations**
3C‐qPCR: chromosome conformation capture followed by quantitative PCR
BG4: G‐quadruplex‐specific antibody clone BG4
G4s: G‐quadruplexes
GEO: gene expression omnibus
HaCaT: immortalized human keratinocyte cells
HKGS: human keratinocyte growth supplement
NHEK: normal human epidermal keratinocytes neonatal
PQS: G4‐seq regions determined under sodium plus pyridostatin
TADs: topologically associating domains
TSS: active transcription start site

G‐quadruplexes (G4s) are distinctive noncanonical DNA secondary structures and have emerged as crucial regulatory elements in nuclear architecture and transcriptional regulation through their association with chromatin remodeling complexes [1, 2]. The human genome harbors numerous putative G4‐forming sequences with nonrandom distribution patterns, enriched in functionally critical genomic regions [3, 4, 5]. Chromatin‐embedded G4 structures identified using G4‐specific antibodies reveal a positive correlation between their presence in promoters and active transcription [1, 6, 7]. While early genome‐wide analyses showed that unfolded G4s are enriched at enhancer elements [8], the role of G4 folding in these regulatory regions and their potential involvement in stabilizing chromatin loops remains poorly understood.

Although computational algorithms and specialized sequencing techniques can predict G4s within DNA sequences [9], their actual formation *in vivo* appears to be predominantly governed by the local chromatin environment. This disparity is exemplified by G4 ChIP‐Seq studies in human cell lines, which demonstrated that only ~ 1% of computationally predicted G4s fold *in vivo* [10]. These folded G4s localize to regulatory, nucleosome‐depleted regions, particularly in promoters and 5′ UTRs of highly transcribed genes, indicating their role in transcriptional regulation and chromatin accessibility.

By recruiting transcription factors and chromatin remodelers, G4 function as structural platforms that organize regulatory complexes at transcriptionally active sites [1]. These DNA structures can additionally act as epigenetic switches, modulating DNA methylation patterns [11]. While some transcription factors preferentially bind to folded G4s through structure‐specific recognition domains [12], others interact with the unfolded G4‐forming sequence [13], potentially influencing G4s formation itself [14]. Recent data revealed that the three‐dimensional G4 structure, rather than the underlying DNA sequence, serves as the primary recognition platform for transcription factors and chromatin modifiers at promoters [15].

The functional importance of G4s is exemplified at the *MYC* locus, where G4s in the promoter positively regulate transcription by altering the chromatin landscape and nucleosome occupancy through preferential binding of transcription factors [15]. Additionally, the downstream *MYC* locus harbors the *PVT1* long noncoding RNA gene [16] that acts as an oncogenic enhancer of *MYC* by facilitating enhancer–promoter interactions [17, 18], enabling distal enhancer elements within the *PVT1* locus to interact with the *MYC* promoter, thereby regulating *MYC* expression.

A pioneering model [19] proposed that folding of G4s facilitates enhancer–promoter interactions through loop formation. This mechanism operates within topologically associating domains (TADs), where CTCF/Cohesin‐mediated chromatin architecture enables spatial proximity between distal regulatory elements and their target promoters [20]. Notably, CTCF is enriched at G4 sites [10], and R‐loops‐associated G4s promote CTCF binding [21, 22, 23], establishing these structures as critical nodes in chromatin loops. Supporting the role in chromatin organization, artificially inserted G4 sequences induced long‐range chromatin modifications [24]. However, whether endogenous G4 folding contributes to regulatory interactions has not been resolved.

Here, we addressed this question by integrating publicly available datasets of G4s distribution, chromatin state, and DNA methylation with locus‐specific chromosome conformation capture (3C‐qPCR) in human keratinocyte cell lines differing in BG4 ChIP‐seq detectable native G4 folding profiles. We found that both folded and unfolded G4s were enriched at active regulatory elements and hypomethylated CpGs, with unfolded G4s uniquely marking weak enhancers. Strikingly, at the *MYC*–*PVT1* locus, enhancer–promoter looping was detected only in the cellular context where detectable G4 folding was present at both interacting regions, suggesting that concurrent detectable G4 folding at both regulatory elements may be associated with enhancer–promoter communication in this context.

## Methods

### Bioinformatic analysis

#### Data acquisition and preprocessing

We used the ChromHMM 18‐state chromatin annotations for Normal Human Epidermal Keratinocytes Neonatal (NHEK) cells generated by Roadmap Epigenomics Consortium [25], which comprise 18 functionally defined chromatin states grouped into promoter‐associated states, transcribed regions, enhancer elements, repressed or silenced domains, and other categories. The data used were obtained from the UCSC Genome Browser for the hg38 assembly.

BG4‐ChIP‐seq data sets for NHEK and spontaneously immortalized human keratinocyte cells (HaCaT) were downloaded from the GEO database (GSE76688) [10], excluding entinostat‐treated samples. We additionally incorporated G4‐seq regions determined under sodium plus pyridostatin conditions (GSE63874) [26] and whole‐genome bisulfite sequencing data for NHEK cells (GSM7445381) [27]. Genomic coordinates originally in hg19 were lifted to hg38 using UCSC liftOver (default parameters). Overlapping intervals within each data source were merged to create nonredundant region sets.

#### Enrichment analysis of NHEK folded and unfolded coordinates in chromatin states

In this study, ‘folded G4s’ were operationally defined as BG4 ChIP‐seq peaks detected in NHEK cells, indicating detectable G4 folding under the experimental conditions used. The term ‘unfolded G4s’ is used in a relative, context‐dependent sense rather than as evidence of absolute structural absence. Specifically, unfolded G4s were evaluated using two complementary approaches. In the first approach, ‘unfolded G4s’ refers to genomic regions with evidence of G4 folding in the closely related keratinocyte cell line HaCaT, but without detectable BG4 enrichment in NHEK. Because the absence of the BG4 signal does not necessarily imply the absence of G4 folding, these sites are interpreted as regions with demonstrated G4‐forming competence in a keratinocyte context, but with no detectable folding signal in NHEK under the conditions tested. This strategy was adopted to enrich loci with demonstrated folding competence while reducing the inclusion of putative G4‐forming regions that may not form stable structures *in vivo* under physiological conditions. In the second approach, ‘unfolded G4s’ were defined as experimentally identified G4‐seq regions determined under sodium plus pyridostatin (PQS) conditions that did not overlap BG4 peaks in NHEK. These loci represent sequences with experimentally demonstrated intrinsic G4‐forming potential *in vitro*, but without detectable BG4 enrichment *in vivo* under the analyzed NHEK conditions. This complementary approach was included to evaluate potential biases introduced by restricting the analysis to BG4‐supported structures while maintaining greater regulatory specificity than purely sequence‐based predictions.

Observed values were calculated by intersecting the genomic coordinates of each G4 group () with the 18 chromatin states using the intersect() function from the pybedtools library [28], yielding 18 values per group. Expected values were obtained through a permutation procedure based on genomic coordinates using the shuffle() function from the pybedtools library. Each group was independently shuffled 1000 times within a set of permissive coordinates, defined based on G4‐seq data obtained under sodium plus pyridostatin conditions. The noOverlapping = True parameter was applied to prevent redundancy among shuffled intervals. Each of the 1000 shuffled sets was then intersected with the chromatin states annotations using the intersect() function from the pybedtools library. The observed values were used as the reference for enrichment analysis and compared against the null distribution derived from the expected values.

#### Enrichment analysis of CpG methylation states in CG sites located in NHEK folded and unfolded G4

An analogous procedure was used to analyze CpG methylation states within folded and unfolded G4 regions. We used a genome‐wide CpG methylation dataset for NHEK [27] restricted to cytosines in the CG context. Based on their beta values, CpG sites were stratified into hypomethylated (< 0.2), partially methylated (0.2–0.8), and hypermethylated (> 0.8) categories. Observed values were calculated by intersecting the genomic coordinates of CpG located within folded and unfolded G4 with the three defined methylation categories, yielding 3 values per group (). Expected values were obtained through a permutation procedure based on genomic coordinates using the shuffle() function from the pybedtools library. Each CG site group was independently shuffled 1000 times within a set of permissive coordinates, defined based on CG coordinates derived from G4‐seq data obtained under sodium plus pyridostatin conditions. The noOverlapping = True parameter was applied to prevent redundancy among shuffled intervals. Each of the 1000 shuffled sets was then intersected with the preclassified genome‐wide CpG methylation dataset using the intersect() function from the pybedtools library. The observed values were used as the reference for enrichment analysis and compared against the null distribution derived from the expected values.

### Cell culture

The same cell lines from the original BG4 ChIP‐seq study were used [10]. HaCaT, a spontaneously immortalized human keratinocyte cell line (RRID:CVCL_0038), authenticated by short tandem repeat (STR) profiling and routinely maintained by our research group, was cultured in Dulbecco's Modified Eagle's Medium (Thermo Fisher Scientific, Waltham, MA, USA) containing 10% fetal bovine serum (Thermo Fisher Scientific) and 1% penicillin and streptomycin (Thermo Fisher Scientific). NHEK, primary normal human epidermal keratinocytes (HEKn pooled, catalog number: A13401; Thermo Fisher Scientific) were cultured in EpiLife™ Medium (cat. no.: M‐EPI‐500‐CA; Thermo Fisher Scientific) supplemented with Human Keratinocyte Growth Supplement (HKGS, cat. no.: S‐001‐5; Thermo Fisher Scientific). Both cell lines were cultured at 37 °C in a humidified atmosphere of 5% CO_2_ and serum‐starved for 24 h before crosslinking to reduce cell‐cycle heterogeneity. All cell lines were confirmed to be mycoplasma‐free using the MycoAlert™ Mycoplasma Detection Kit (LT07‐318; Lonza), and only mycoplasma‐negative cultures were used in all experiments.

### Chromosome conformation capture (3C)‐qPCR

3C‐qPCR was performed as previously described [29] with modifications for keratinocytes. Target regions were selected based on BG4 ChIP‐seq data [10], screening chr8:127 000 000–129 000 000 using ENCODE enhancer markers for NHEK cells. Enhancer regions E1–E3 were defined based on NHEK ChromHMM annotations, supported by H3K4me1 and H3K27ac enrichment.

The 3C protocol was optimized to identify the most efficient conditions for digestion using the selected restriction enzyme. A published protocol for 4C using keratinocyte cells with greater resistance to lysis was adapted [30]. This protocol evaluates lysis efficiency indirectly using trypan blue and light microscopy. Additional lysis steps were introduced if the cytoplasm remained intact, resulting in an overall digestion efficiency of ~ 95%.

Cells were fixed with 1% formaldehyde in PBS. Nuclear extraction used lysis buffer with Dounce homogenization (30 strokes), verified by trypan blue staining. Nuclear permeabilization was achieved with SDS (final concentration 0.3%). After testing different enzyme concentrations and incubation times, serial enzyme addition of HindIII enzyme yielded the highest nuclei digestion. Instead of adding 450 U of the enzyme at once for overnight digestion, 150 U were added initially, followed by 2 h of incubation. An additional 150 U were added for another 2 h of incubation, and finally, 100 U were added for overnight incubation with agitation. To assess digestion efficiency, qPCR was performed on digested and undigested DNA aliquots using primers that amplify across restriction sites. To correct differences in the template amount, the amplification of a control region without restriction sites was used for normalization.

Restriction enzyme inactivation was carried out using SDS, with the temperature reduced from 65 to 55 °C to prevent crosslink reversal. Subsequent steps, including ligation, crosslink reversal, and DNA purification, followed previously established protocols in our laboratory.

PCR was performed on all restriction fragments covering the locus of interest using preselected primers. PCR products were purified and quantified, pooled in equimolar amounts and digested with the same restriction enzyme used for the 3C library. The fragments were ligated to create all possible interactions within the locus. This control library was used to test primers and normalize 3C‐qPCRs, accounting for the efficiency of each primer pair. A pair of primers without a restriction site between them was included as a loading control.

3C interaction products were detected using SYBR Green qPCR with selected anchor and bait primer pairs (Table S1). Primers were designed to be longer (~ 25 bp) to minimize nonspecific amplification in the low‐target samples. The qPCR reactions were performed with PowerUp SYBR Green Master Mix (Thermo Fisher Scientific, USA), 200 nm of anchor primer and bait/control primers, and 50 ng of 3C libraries (quantified by qPCR). All samples were tested in triplicate. qPCR conditions were as follows: initial denaturation at 95°C for 3 min, followed by 40 cycles of 95 °C for 15 s and 60 °C for 1 min for annealing and extension.

3C‐qPCR results were normalized to a serial dilution standard curve from the control library on each plate and for each primer pair. In 3C‐qPCR, raw ligation frequencies must be normalized to correct for differences in amplification efficiency between primer pairs targeting different restriction fragments. To this end, a PCR‐based control ligation library was constructed as follows: each restriction fragment spanning the *MYC*–*PVT1* locus analyzed by 3C was individually amplified by PCR using the same primers employed in the 3C assay. The purified PCR products were mixed in equimolar amounts, digested with the same restriction enzyme used for 3C library preparation, and subjected to random intermolecular ligation under dilute conditions. This generates a reference library in which all possible ligation products are present at equivalent molar concentrations, independent of chromatin structure or cellular context. Interaction frequencies were then calculated as the ratio of the qPCR signal in the 3C library to the signal in the control library for each primer pair, thereby normalizing for any differences in primer amplification efficiency across the locus. To mimic 3C sample conditions, the control library concentration was adjusted by adding digested and randomly ligated genomic DNA to increase complexity and prevent PCR efficiency bias from total DNA concentration. Final values were calculated using the equation: value = 10 (*C*_t_ − *b*)/*a* (where *b* is the intercept and *a* is the slope). These values were further normalized to an internal control (*GAPDH*).

*MYC* transcript levels were assessed by quantitative RT‐PCR. Total RNA was isolated from HaCaT and NHEK cells using TRIzol reagent following manufacturer's instructions. One microgram of RNA was reverse‐transcribed using SuperScript IV reverse transcriptase with oligo(dT) primers. Real‐time PCR was performed using the following MYC‐specific primers: forward 5′‐TGAGGAGACACCGCCCAC‐3′ and reverse 5′‐AACATCGATTTCTTCCTCATCTTC‐3′. GAPDH served as the reference gene. Reactions were performed in triplicate using PowerUp SYBR Green Master Mix under standard cycling conditions. Relative expression was determined using the $2^{-\Delta \Delta C_{t}}$ method, with HaCaT as the reference sample.

### Statistical analysis

After performing the permutation procedure, we assessed the normality of the distribution of intersection counts from the shuffled datasets using Q–Q plots.

The Z‐score of each observed value was calculated by subtracting the mean of the 1000 permuted values from the observed values and dividing the result by the standard deviation of the expected values. For visual comparison, the expected values from the permutations were also standardized using the same parameters.

To determine which observed Z‐scores were statistically significant relative to their corresponding null distributions, we first computed two‐tailed *P*‐values from the standard normal distribution using the absolute Z‐scores and the cumulative distribution function. We then applied Bonferroni correction to account for multiple hypothesis testing by multiplying each raw *P*‐value by the number of tested categories. Results were considered significant if Bonferroni‐adjusted *P*‐value ≤ 0.01. The corresponding critical Z‐score was obtained with the ppf() function from scipy.stats.norm, using two‐tailed Bonferroni *a* = 0.01 cutoff. Because the null distributions were standardized, the function was applied with its default parameters (mean 0, standard deviation 1).

Statistical significance for 3C‐qPCR interaction frequencies was assessed using the Kruskal–Wallis test followed by Bonferroni correction for three planned enhancer–promoter comparisons between the *MYC* promoter bait and the distal regions E1, E2, and E3. For each anchor–bait pair, contact frequencies were quantified in three independent biological replicates, using adjacent fragments as local background controls. A corrected *P*‐value < 0.05 was considered statistically significant. Differences in MYC expression between HaCaT and NHEK cells were assessed using an unpaired two‐tailed Student's *t*‐test applied to Δ*C*_t_ values, with *P* < 0.05 considered statistically significant.

All analyses were performed using Python (version 3.11) and r (version 4.4.1), with standard statistical and visualization packages.

## Results

### Genome‐wide distribution of G4 structures is enriched in active regulatory elements

**Fig. 1:**
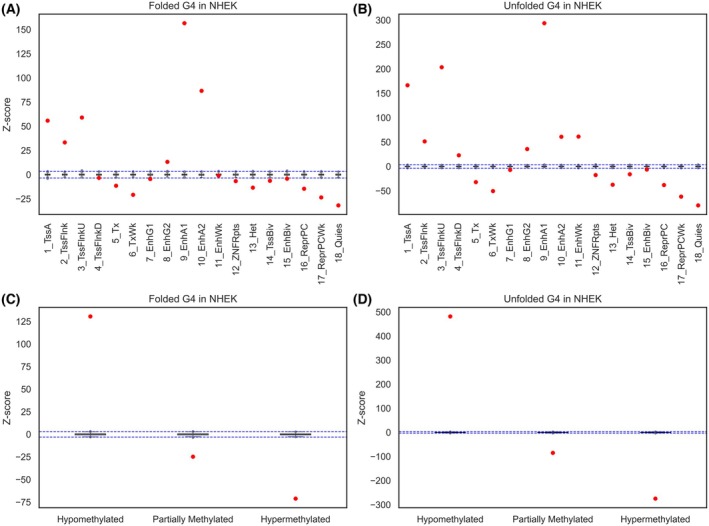
Enrichment of folded and unfolded G‐quadruplexes (G4s) across chromatin states and CpG methylation states. (A) Folded G4s, defined as G‐quadruplex‐specific antibody clone BG4 (BG4 ChIP‐seq) peaks detected in primary normal human epidermal keratinocytes neonatal (NHEK). (B) Unfolded G4s in NHEK, defined as BG4 ChIP‐seq peaks present in immortalized human keratinocytes (HaCaT) but absent in NHEK. Red points indicate observed Z‐scores for each chromatin state; gray boxes show permutation Z‐score distributions (1000 shuffles) for NHEK folded and unfolded G4 coordinates, with shuffling confined to G4‐seq regions determined under sodium plus pyridostatin. Blue dashed lines correspond to the two‐tailed Bonferroni significance threshold at *α* = 0.01; points above or below these thresholds indicate significant enrichment or depletion. Chromatin state labels: 1_TssA, active transcription start site (TSS); 2_TssFlnk, flanking TSS; 3_TssFlnkU, upstream TSS flanking; 4_TssFlnkD, downstream TSS flanking; 5_Tx, strong transcription; 6_TxWk, weak transcription; 7_EnhG1 and 8_EnhG2, genic enhancers; 9_EnhA1 and 10_EnhA2, active enhancers; 11_EnhWk, weak enhancer; 12_ZNF/Rpts, zinc finger genes and repeats; 13_Het, heterochromatin; 14_TssBiv, poised TSS; 15_EnhBiv, bivalent enhancer; 16_ReprPC, Polycomb‐repressed; 17_ReprPCWk, weakly Polycomb‐repressed; 18_Quies, quiescent/low signal. (C) Folded G4s in NHEK, as defined above, analyzed for enrichment across CpG methylation states. (D) Unfolded G4s in NHEK, as defined above, analyzed for enrichment across CpG methylation states. CpGs were classified as hypomethylated (*β* < 0.2), partially methylated (0.2 ≤ *β* ≤ 0.8), or hypermethylated (*β* > 0.8). Red points indicate observed Z‐scores for each category; gray boxes represent Z‐score distributions from 1000 coordinate permutations. Blue dashed lines correspond to the two‐tailed Bonferroni significance threshold at *α* = 0.01; points above or below these thresholds indicate significant enrichment or depletion.

We explored the genomic landscape of G4 structures across the 18 chromatin states defined by ChromHMM in NHEK cells. To investigate differences in G4 detection across keratinocyte cell types, we analyzed two categories of G4 sites. Folded G4s were defined as BG4 ChIP‐seq peaks detected in NHEK cells, whereas unfolded G4s corresponded to peaks detected in the closely related keratinocyte cell line HaCaT but not detected in NHEK (see Methods for operational definitions). Using these criteria, we identified 2506 folded G4 peaks and 17 012 unfolded G4 peaks genome‐wide, indicating a substantial difference in BG4‐detectable G4 landscapes between the two cell types. Because enrichment was evaluated independently for each group using permutation tests that preserve the number of intervals, differences in set size do not bias the analysis, and enrichment was interpreted relative to the null distribution of each dataset rather than by direct comparison of absolute counts between groups. Our enrichment analysis revealed that both folded G4s (Fig. 1A) and unfolded G4s (Fig. 1B) were significantly enriched at active enhancers and TSS regions. Interestingly, only unfolded G4s exhibited significant enrichment at weak enhancers, suggesting a potential regulatory distinction between the two G4 categories. Both groups were strongly depleted from heterochromatin and Polycomb‐repressed domains, consistent with the preferential localization of G4 structures to regulatory and transcriptionally active chromatin regions [1, 6]. These observations are consistent with a model in which unfolded G4s represent sequences with G4‐folding competence that are detectable in HaCaT but not in NHEK, potentially reflecting differences in chromatin accessibility or regulatory context. In contrast, folded G4s tend to occur at regulatory elements consistently detected across both keratinocyte cell types.

To evaluate whether restricting the analysis to BG4‐supported G4 structures could introduce bias in the identification of regulatory elements, we performed a complementary permutation analysis, including all PQSs within the same genomic intervals (Fig. S1). PQSs were broadly enriched across open chromatin regions, including active promoters and enhancer‐associated states, consistent with the well‐established association between G4‐forming potential and GC‐rich regulatory DNA. However, two key differences emerged. First, PQSs did not recapitulate the selective enrichment of unfolded G4s at weak enhancer states (states 8_EnhG1 and 9_EnhA1), suggesting that this regulatory distinction is more clearly resolved with BG4 analyses. Second, the inclusion of all PQSs substantially increased the number of candidate loci (*N* = 715 456 nonredundant genomic loci) without a corresponding gain in regulatory specificity, as many G4‐forming regions identified by G4‐seq may form G4s *in vitro* but not exhibit detectable G4 formation in keratinocytes under the analyzed conditions. For this reason, subsequent analyses focused on BG4‐supported G4s, which provide experimental evidence of G4 formation in keratinocytes under the analyzed conditions.

### Folded and unfolded G4 regions are associated with hypomethylated CpGs

We examined DNA methylation patterns at folded and unfolded G4 regions using bisulfite sequencing data from NHEK cells. Both G4 categories were highly enriched for hypomethylated CpGs (0–20% methylation), consistent with their localization in open chromatin (Fig. 1C). Conversely, G4‐containing regions were depleted of partially methylated and hypermethylated CpGs (Fig. 1D). These results indicate that G4 sequence potential, rather than BG4 detection status, is associated with maintained hypomethylation at these genomic regions.

### Folded G4s is associate with increased enhancer–promoter interactions at the *MYC* – *PVT1* locus

**Fig. 2:**
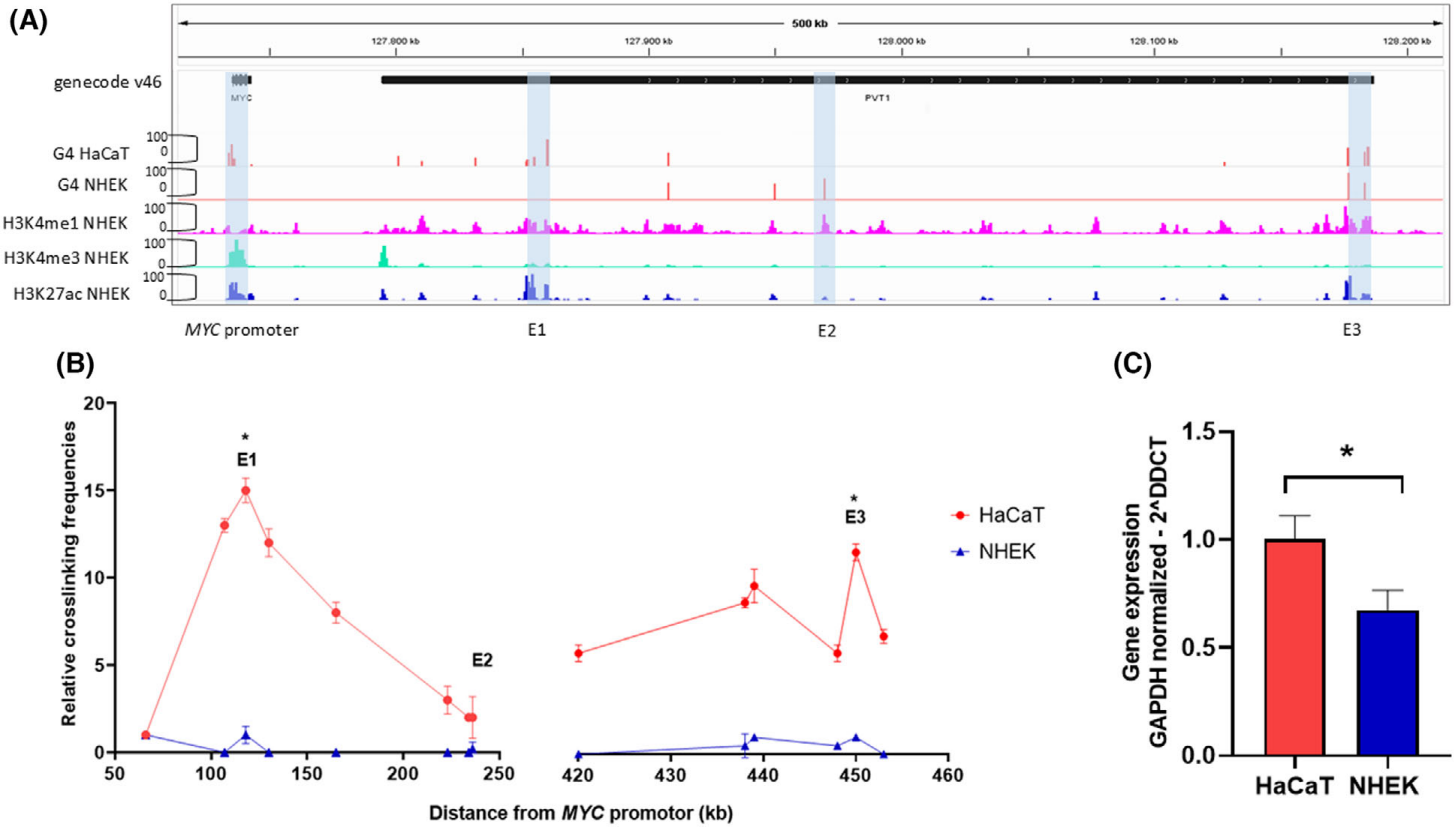
Detectable G‐quadruplexe (G4) folding is associated with enhancer–promoter looping at the *MYC*–*PVT1* locus. (A) Genome browser view of chromosome 8 (hg38: 127.8–128.2 Mb) showing GENCODE v46 gene annotations, G‐quadruplex‐specific antibody clone BG4 (BG4 ChIP‐seq) peaks in immortalized human keratinocytes (HaCaT) and primary normal human epidermal keratinocytes neonatal (NHEK), and NHEK histone modification tracks (H3K4me1, H3K4me3 and H3K27ac) derived from publicly available datasets generated by the Roadmap Epigenomics Consortium. All ChIP‐seq tracks are shown using identical scaling to allow direct visual comparison between conditions. The *MYC* promoter and three distal enhancers (E1–E3) within the *PVT1* locus analyzed in this study are highlighted in blue. (B) Relative crosslinking frequencies between the MYC promoter and distal fragments measured by chromosome conformation capture (3C‐qPCR) in HaCaT (red) and NHEK (blue) cells. Data are presented as mean ± SD of *n* = 3 biological replicates, normalized to a control fragment. HaCaT cells exhibit increased interaction frequencies between the MYC promoter and enhancers E1 and E3, whereas NHEK cells show only background interactions; asterisks indicate *P* < 0.05, Kruskal–Wallis test followed by Bonferroni correction. (C) Relative MYC expression in HaCaT and NHEK cells. Data are presented as mean ± SD of *n* = 3 biological replicates. Asterisks indicate *P* < 0.05, unpaired two‐tailed Student's *t*‐test applied to Δ*C*_t_ values.

To investigate the functional significance of folded G4s in enhancer–promoter communications, we examined the well‐characterized *MYC*‐*PVT1* locus. Comparative analysis of BG4 ChIP‐seq data revealed distinct G4 landscapes between HaCaT and NHEK cells. HaCaT cells displayed prominent G4 peaks at the *MYC* promoter and enhancer regions E1 and E3 (H3K4me1 and H3K27ac) within the *PVT1* locus. In contrast, NHEK cells exhibited G4 enrichment at E2 and E3, with absent signal at the *MYC* promoter and E1 (Fig. 2A). Based on this differential BG4 detection profile, we asked whether enhancer–promoter interaction frequencies would differ between the two cell lines. We hypothesized that these interactions at the *MYC* locus would be more frequent in HaCaT, where folded G4s were present at both regulatory elements. Using 3C‐qPCR analysis across the *MYC*‐*PVT1* locus in the two cell lines, we found significantly higher interaction frequencies between the *MYC* promoter and its enhancers in HaCaT cells compared with NHEK cells. Specifically, enhancers E1 and E3 showed significantly higher interaction with the *MYC* promoter in HaCaT cells relative to their neighboring genomic regions (*P* < 0.05), while E2, despite containing folded G4s in both cell types, showed no significant interaction when the *MYC* promoter lacked folded G4s (Fig. 2B). Importantly, the presence of canonical enhancer marks (H3K4me1 and H3K27ac) at E3 in NHEK cells was not associated with detectable enhancer–promoter looping when BG4 signal was absent at the MYC promoter. Consistent with the increased looping, *MYC* transcript levels were significantly higher in HaCaT compared with NHEK (Fig. 2C). These findings indicate that detectable enhancer–promoter looping at the *MYC*–*PVT1* locus is observed in the context of folded G4s at both interacting elements, while permissive chromatin marks alone were not associated with looping.

**Fig. 3:**
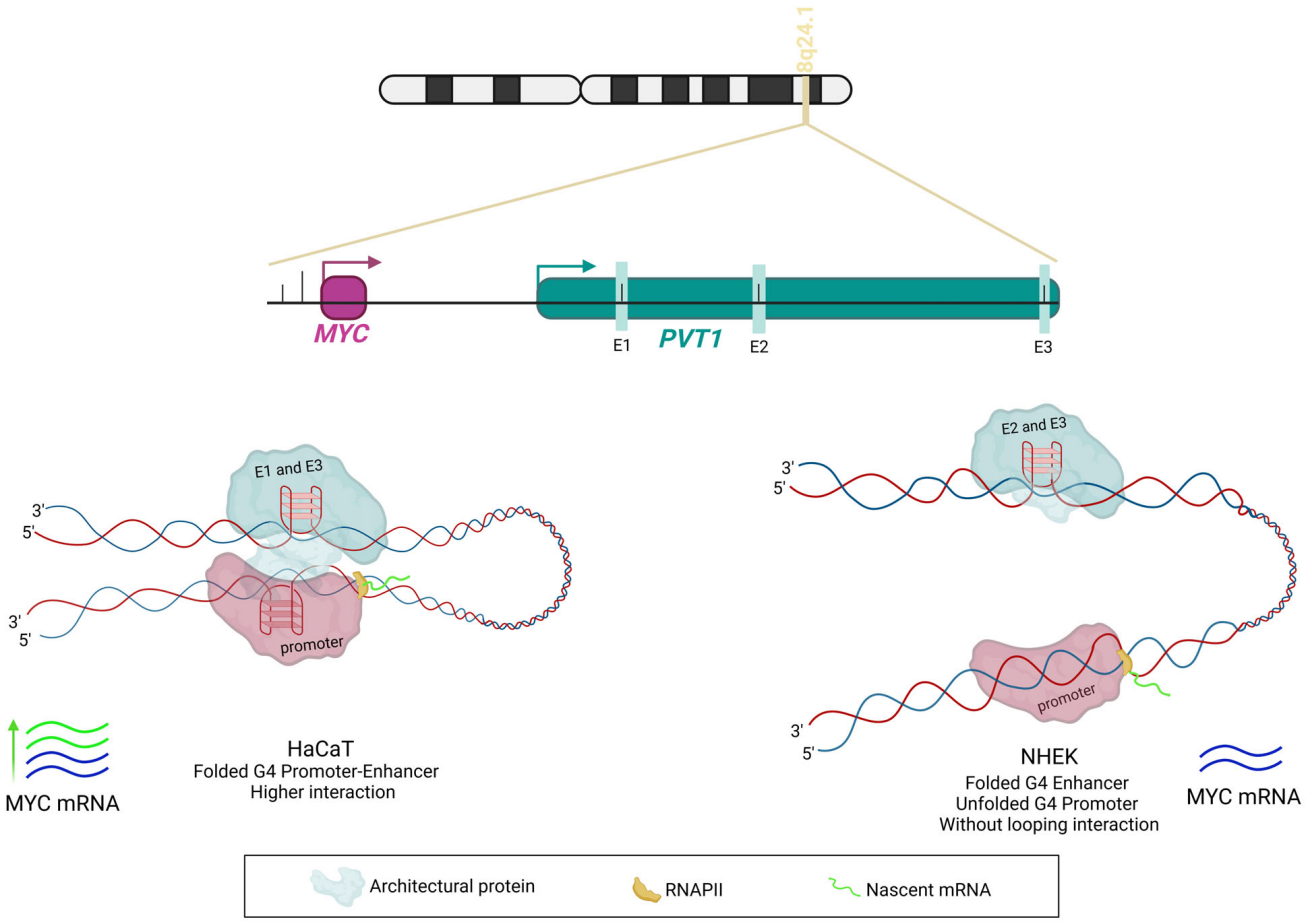
Suggested model for dual G‐quadruplexe (G4) folding in enhancer–promoter communication. Schematic representation of the *MYC*–*PVT1* locus showing that looping between the *MYC* promoter and distal enhancers (E1 and E3) is observed in HaCaT cells when native folded G4 structures are present at both regions, associated with higher *MYC* expression. In contrast, in NHEK cells, the absence of BG4‐detectable G4 folding at the *MYC* promoter coincides with a lack of detectable looping and lower *MYC* expression. The model highlights the concurrent presence of detectable G4 folding at both regulatory elements in association with enhancer–promoter communication. Created in biorender. De Lima, D. (2026) https://BioRender.com/xme0nvx.

Consistent with the differential BG4 profiles, a prominent BG4 signal was detected at the *MYC* promoter in HaCaT but was not detected in NHEK (Fig. 3A), paralleling the higher basal MYC expression observed in HaCaT.

## Discussion

Our study reveals an association between concurrent BG4‐detectable G4 folding at enhancer and promoter elements and enhancer–promoter communication at the *MYC* locus. By integrating genome‐wide chromatin state, DNA methylation, and G4 mapping with locus‐specific 3C‐qPCR, we compared keratinocyte cell lines that share similar chromatin landscapes but differ in G4 folding profiles. At the *MYC*–*PVT1* locus, chromosomal looping was detected in the cellular context where folded G4s were present at both promoter and enhancer regions, whereas active histone marks alone were not associated with detectable interactions. This finding extends the functional repertoire of G4s beyond promoter‐proximal regulation and provides additional support for the hypothesis that native DNA secondary structures contribute to three‐dimensional genome organization.

Our genome‐wide analysis revealed distinct distribution patterns for the BG4‐detectable G4 folding categories examined in this study. Both folded and unfolded G4s were enriched at transcription start sites (TSS), active enhancers, and hypomethylated CpGs, consistent with an open chromatin configuration. However, unfolded G4s exhibited unique enrichment at weak enhancers, a chromatin state typically associated with lower transcriptional output and regulatory plasticity. These observations suggest that unfolded G4s may mark regulatory elements with characteristics consistent with a poised or weakly activated state, whereas folded G4s were more frequently associated with active regulatory elements. This framework aligns with CUT&Tag findings [31] showing G4s in both active and poised enhancers, suggesting that G4 folding may be associated with specific enhancer activity states and potentially influence enhancer–promoter interactions.

The *MYC*–*PVT1* locus represents a well‐established model of long‐range enhancer–promoter regulation and provides an endogenous context to examine the contribution of G4 folding to chromatin looping. The concurrent BG4‐detectable folding pattern we observe aligns conceptually with the intermolecular G4 tether model [19], which proposes that complementary G4‐forming sequences in distal elements could pair with proximal promoters to facilitate loop stabilization. In this framework, folded G4s may represent a layer that complements, rather than substitutes for, canonical chromatin features. Importantly, this pattern is observed here at an endogenous locus and under native chromatin conditions, without artificial sequence insertion or targeted genome manipulation. The finding that active histone marks alone were not sufficient to explain looping in our system further suggests that BG4‐detectable folding at both regulatory anchors may be associated with detectable long‐range communication. Notably, while enhancer‐associated G4s may establish a permissive regulatory environment, the absence of detectable BG4 enrichment at the promoter may represent a feature associated with reduced enhancer–promoter looping in this context.

Previous studies showed the importance of G4 folding in gene regulation. Disruption of enhancer G4 folding by genome editing impairs chromatin accessibility, RNA polymerase II recruitment, and enhancer–promoter contacts at the globin loci [32], effects comparable to complete enhancer deletion. Conversely, restoring G4 folding with an alternative sequence reinstated these features, showing that structure, not sequence, is the functional determinant. At the *MYC* promoter, G4 folding similarly regulates transcription by modulating nucleosome occupancy and transcription factor recruitment independent of DNA sequence [15]. In addition, insertional experiments of *de novo* G4s [24] and genome‐wide analyses of endogenous G4s [33] have shown that these structures are associated with long‐range chromatin interactions and can induce enhancer‐like histone modifications, supporting a role for G4s in three‐dimensional genome architecture. However, these studies did not resolve whether G4 folding at a single regulatory element is sufficient to support enhancer–promoter communication under native chromatin conditions. By contrast, our analysis of the endogenous *MYC*–*PVT1* locus, which preserves sequence context and epigenetic state, reveals that looping was detected only in the context of simultaneous G4 folding at enhancer and promoter sites, with looping observed preferentially when detectable BG4 enrichment was present at both interacting regions in the *MYC*–*PVT1* context examined here.

We also note that *MYC* expression is higher in HaCaT compared with NHEK cells, and increased transcriptional activity has been associated with elevated chromatin interaction frequencies [34] thus, transcriptional output could contribute to the interaction patterns observed here. However, promoter activity alone does not fully explain the looping pattern detected at this locus. In HaCaT cells, the E2 enhancer lacks detectable BG4 signal and does not exhibit increased interaction with the *MYC* promoter despite higher *MYC* expression. Conversely, in NHEK cells, distal enhancers display active histone marks but do not show detectable promoter contacts when BG4 signal is absent at one of the interacting elements. Therefore, while transcriptional activity may modulate contact frequency, detectable looping at the *MYC*–*PVT1* locus is most consistently observed when BG4‐detectable G4 folding is present at both regulatory anchors.

While HaCaT and NHEK are both keratinocyte‐derived and share a common phenotypic origin, immortalization and culture conditions may impose subtle differences in chromatin organization, potentially influencing G4 folding or looping patterns. In addition, our 3C‐qPCR analyses were confined to the *MYC*–*PVT1* region, and further studies across additional loci and cell types will be essential to establish the generality of the concurrent folding model. Finally, because BG4 ChIP‐seq detects G4 structures that are accessible and sufficiently stable for antibody recognition, the absence of BG4 signal does not necessarily indicate absence of G4 folding. Accordingly, the folded and unfolded categories used in this should be interpreted as reflecting differential BG4 detection across cellular contexts rather than definitive structural states.

In conclusion, our results support a model in which simultaneous native G4 folding detectable by BG4 ChIP‐seq at enhancers and promoters is associated with chromosomal looping at the *MYC* locus. (Fig. 3). This observation distinguishes our findings from previous single‐site G4 studies and suggests that coordinated G4 folding at both regulatory elements is associated with enhancer–promoter communication in contexts where canonical chromatin marks alone were not sufficient for detectable looping. Together, these findings position G4 structures not only as markers of active chromatin but also as features associated with enhancer–promoter interactions.

## Conflict of interest

The authors declare no conflict of interest.

## Author contributions

DGL and GNG performed cell culture experiments and 3C‐qPCR assays. EOSA conducted the bioinformatic analyses. DGL, GNG, and EOSA contributed to manuscript editing. BCFB assisted with the 3C‐qPCR experiments. MJ‐E prepared the datasets for bioinformatic analysis. SRPL and APS contributed to discussion and manuscript editing. ACP conceptualized and supervised the study, organized the project, and wrote the manuscript.

## Supporting information


**Fig. S1.** Complementary PQS‐based enrichment analysis across chromatin and DNA methylation states. (A, B) Z‐score distribution of G4‐seq‐supported loci (PQSs) enrichment across the 18‐state ChromHMM annotation in NHEK cells. G4‐seq‐supported loci were broadly enriched in open and regulatory chromatin states, including transcription start site‐associated and enhancer states, consistent with the known association between G4‐forming potential and GC‐rich regulatory DNA. (C, D) Enrichment of G4‐seq‐supported loci across CpG methylation categories in NHEK cells, classified as hypomethylated, partially methylated, or hypermethylated. Red points indicate observed Z‐scores for each chromatin or methylation state. Blue dashed lines indicate the enrichment/depletion threshold used to identify significant deviations from the permutation‐derived null distribution. Unlike the BG4‐based analysis, the G4‐seq‐supported G4‐forming loci‐based analysis showed broad enrichment across regulatory regions but did not recapitulate the selective enrichment pattern observed for BG4‐supported unfolded G4s at weak enhancer states, supporting the use of experimentally detected G4 structures for the main analyses.
**Table S1.** Primers for 3C‐qPCR.

## Data Availability

All data supporting the findings of this study are available within the paper and its Supporting Information files. Genome‐wide datasets used for G4 mapping, chromatin state annotation, and DNA methylation analyses are publicly available in the Gene Expression Omnibus (GEO) under accession codes GSE76688, GSE63874, and GSM7445381. Primers for 3C‐qPCR interaction frequency data for the *MYC*–*PVT1* locus are provided in Table S1. Additional raw data generated in this study are available from the corresponding author upon reasonable request.
