# Supplementary material for: Dual native G‐quadruplex folding is associated with chromatin looping at the MYC locus
Source: FEBS Open Bio. 2026 Jul 23:10.1002/2211-5463.70316. Online ahead of print. doi: 10.1002/2211-5463.70316 (PMC13393508; doi:10.1002/2211-5463.70316)
Supplement: Supplementary file 1 — Fig. S1. Complementary PQS‐based enrichment analysis across chromatin and DNA methylation states. (A, B) Z‐score distribution of G4‐seq‐supported loci (PQSs) enrichment across the 18‐state ChromHMM annotation in NHEK cells. G4‐seq‐supported loci were broadly enriched in open and regulatory chromatin states, including transcription start site‐associated and enhancer states, consistent with the known association between G4‐forming potential and GC‐rich regulatory DNA. (C, D) Enrichment of G4‐seq‐supported loci across CpG methylation categories in NHEK cells, classified as hypomethylated, partially methylated, or hypermethylated. Red points indicate observed Z‐scores for each chromatin or methylation state. Blue dashed lines indicate the enrichment/depletion threshold used to identify significant deviations from the permutation‐derived null distribution. Unlike the BG4‐based analysis, the G4‐seq‐supported G4‐forming loci‐based analysis showed broad enrichment across regulatory regions but did not recapitulate the selective enrichment pattern observed for BG4‐supported unfolded G4s at weak enhancer states, supporting the use of experimentally detected G4 structures for the main analyses. Table S1. Primers for 3C‐qPCR. [file FEB4-9999-0-s001.docx]

Supplementary Table 1- Primers for 3C-qPCR

| Primer name | Restriction enzyme site on chr 8 | Distance to MYC promoter (bp) | Primer F | Primer R |
| --- | --- | --- | --- | --- |
| Anchor | 127733744 | promoter | TAGCTCCTTCAGGAGAGAACTTACA | CATGAACACTTGTTTCCCTCAACTCT |
| bait control 1 | 127800353 | 66609 | ATTTCCTGAGCAGTTGCTGTATGC | ACTTTGCACAGGAGTTAGCTTTTGC |
| bait control 2 | 127841031 | 107287 | TGTTCAGACAGCTTCCAGTTCTG | CCCTCTGATGTATACTTTCCCCATT |
| E1 | 127852350 | 118606 | TTGTGTTTACTTTGTGTGGGATTCC | GAATGACTTGCCCAGAAGAGTTTG |
| bait control 3 | 127864101 | 130357 | GATGGAGCGAGATGTAGGGGAAA | CTTCACCCCACGTAACCTTTCTTTC |
| bait control 4 | 127899104 | 165360 | TAAACAAATAAACACTGGCTGGCC | GGGATTTTCTAACATGCCTCAACA |
| bait control 5 | 127956873 | 223129 | TTCTCTGCAAAACTTGAAAACAGGG | CATTAAACCACCATGCAAGCTAGG |
| bait control 6 | 127968493 | 234749 | ATCTGGATGCCTGTTGAGTGTAG | GCCTCTCATTGTGTCTTCAGTTAAC |
| E2 | 127969882 | 236138 | TTGCCTAAGGTCTACACAGTTTACC | TGTCTTCTTGTCTTCTTTTCTGGGA |
| bait control 7 | 128152994 | 419250 | CCATGGGCTACTCTCTTCATATACC | ATGTCCAGCTGTCATCTGTGTTTA |
| bait control 8 | 128172315 | 438571 | TATTATTGAGCAGACAGGTGGATGA | TTCCCCTATTCATTCTTCCATCAGA |
| bait control 9 | 128173628 | 439884 | GGCAGGAATCCTCTTGACATTCTAA | GGATCATTCTGGCTAAAGGAGACAA |
| bait control 10 | 128182011 | 448267 | GCAAAGCCACTCTGGAAGACAATAC | CTCAGAAGGTGGAGTTCATGTTCCT |
| E3 | 128183944 | 450200 | GCTTATTTATTTAATTGATTGGCTTTC | TAGACAGTAGGGACACAGCAGTGGA |
| bait control 11 | 128187509 | 453765 | AAGCCTTTCTGTTCCACTGTGTCTC | CATAGCTTCAGCAATTTGGCTTCTT |


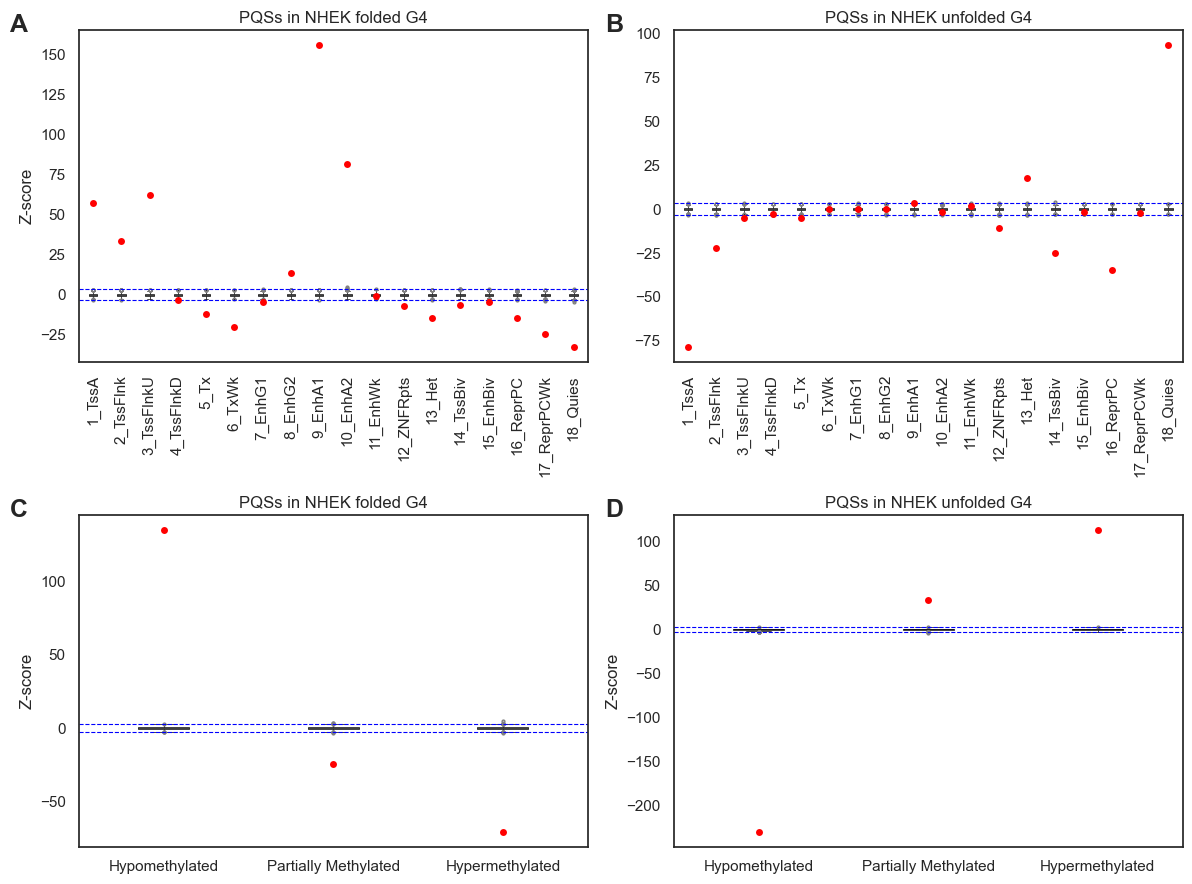


**Supplementary Figure 1. Complementary PQS-based enrichment analysis across chromatin and DNA methylation states.** (A, B) Z-score distribution of G4-seq-supported loci (PQSs) enrichment across the 18-state ChromHMM annotation in NHEK cells. G4-seq-supported loci were broadly enriched in open and regulatory chromatin states, including transcription start site-associated and enhancer states, consistent with the known association between G4-forming potential and GC-rich regulatory DNA. (C, D) Enrichment of G4-seq-supported loci across CpG methylation categories in NHEK cells, classified as hypomethylated, partially methylated, or hypermethylated. Red points indicate observed Z-scores for each chromatin or methylation state. Blue dashed lines indicate the enrichment/depletion threshold used to identify significant deviations from the permutation-derived null distribution. Unlike the BG4-based analysis, the G4-seq-supported G4-forming loci-based analysis showed broad enrichment across regulatory regions but did not recapitulate the selective enrichment pattern observed for BG4-supported unfolded G4s at weak enhancer states, supporting the use of experimentally detected G4 structures for the main analyses.
